# Bidirectional Photoadaptive Organic Heterojunction Synaptic Transistors for Accurate Image Recognition in Machine Vision Systems

**DOI:** 10.1002/advs.202517059

**Published:** 2025-11-19

**Authors:** Di Xue, Hongyu Liu, Yingying Zhang, Feng Ding, Jie Lu, Yao Yin, Zi Wang, Jianlong Xu, Lifeng Chi, Lizhen Huang

**Affiliations:** ^1^ Institute of Functional Nano and Soft Materials (FUNSOM) State Key Laboratory of Bioinspired Interfacial Materials Science Jiangsu Key Laboratory for Carbon‐Based Functional Materials and Devices Soochow University Suzhou 215123 China; ^2^ Suzhou Laboratory Suzhou 215123 China

**Keywords:** bidirectional photoconductivity, image recognition, neuromorphic synapses, organic heterojunctions, photoadaptation

## Abstract

Machine vision systems are crucial in intelligent scenarios, but actual image acquisition is frequently compromised by the inadequate proficiency of photosensors in photoadaptation. Inspired by biological vision, neuromorphic synaptic phototransistors endowed with photoadaptive capabilities have emerged as a prospective strategy. However, most synaptic phototransistors only exhibit unidirectional positive photoresponses, whereas those capable of bidirectional photoresponses offer a greater possibility of accurately capturing images in complex lighting scenes. Herein, bidirectional photoadaptable organic heterojunction synapse phototransistors as sensing and processing units in systems are reported, which facilitate image contrast enhancement and improve image feature extraction under adverse lighting conditions. The bidirectional plasticity transformation of biomimetic neuromorphic synapses is mimicked. Specifically, n–n heterojunctions exhibit a unidirectional excitatory postsynaptic current, whereas n‐p heterojunctions show a bidirectional response with a more prominent inhibitory postsynaptic current. Most interestingly, by integrating the device characteristics into convolutional neural networks and simultaneously optimizing algorithm architecture, the details and edges of low‐contrast images are markedly enhanced, and the accuracy of image recognition is increased to 97.4% within ten cycles. This work serves as a novel idea for the development of high‐performance neuromorphic visual systems, rendering them promising candidates for in‐sensor computing applications.

## Graphical abstract

Bidirectional photoadaptive organic synaptic phototransistors are developed using complementary n–n and n‐p heterojunctions. These devices enable intelligent in‐sensor processing, markedly enhancing image contrast and edge features under adverse lighting, and achieve a recognition accuracy of 97.4% within ten training cycles for machine vision.

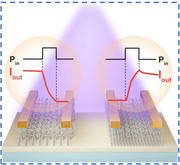

## 1 Introduction

Machine vision systems, analogous to human vision, consist of image acquisition, preprocessing, and analysis templates, enabling crucial functions such as detection, processing, and recognition.^[^ ^1^, ^2^, ^3^ ^]^ They serve as the eyes of intelligent applications and play a pivotal role in autonomous driving, robotics, industry manufacturing and other various intelligent scenarios.^[^ ^4^, ^5^, ^6^ ^]^ However, the acquisition of real‐world image data often presents unstructured challenges, including uneven illumination and unstable fields of view. When optical sensors lack sufficient adaptability, the captured images can become blurred or distorted, significantly degrading the recognition performance. While hardware solutions such as specialized lighting equipment or automatic dimming systems can mitigate these issues, they increase system design and manufacturing costs, often leading to increased power consumption and maintenance expenses. To streamline the hardware architecture of the system while improving the efficiency and accuracy of image recognition, precise in situ methods can be used to process irregular information in images for image enhancement and filtering.^[^ ^7^, ^8^ ^]^

Photoadaptive devices equipped with intelligent in‐sensor processing regulation systems can dynamically respond to ambient light variations in real time. Their dynamic modulation mechanisms closely resemble the adaptive regulation of biological retinas, offering a novel solution to the light adaptability challenges faced by conventional optoelectronic devices.^[^ ^9^, ^10^, ^11^, ^12^, ^13^, ^14^ ^]^ Organic phototransistors (OPTs), typical examples of neuromorphic synaptic devices, can simulate the excitatory and inhibitory processes of synapses, which fundamentally aligns with the core concept of photoadaptive regulation.^[^ ^15^, ^16^, ^17^, ^18^ ^]^ However, most current organic phototransistor synaptic devices exhibit only a unidirectional positive photoconductivity (PPC) response.^[^ ^19^, ^20^, ^21^, ^22^, ^23^ ^]^ Limited by insufficient sensitivity and detection capability under weak‐light conditions, these devices might suffer from inefficient extraction of effective optical signals, rendering them ill‐suited for complex lighting scenarios. Bidirectional photoconductive devices, which combine positive and negative photoconductivity (PPC/NPC) effects, offer unique advantages in terms of photoadaptive regulation by mimicking the excitatory‐inhibitory balance mechanism of biological vision. In recent years, various material systems ranging from 2D materials, metal oxides, halide perovskites to organic semiconductors combined with device structural design or interface engineering has significantly accelerated the progress, enabling controllable bidirectional photoresponse and enhanced photoadaptive performance, and thereby advancing the development of highly efficient in‐sensor functionalities.^[^ ^17^, ^24^, ^25^ ^]^ Among them, bidirectional photoresponsive synaptic transistors based on organic semiconductor heterostructure have gained particular attention for constructing low‐cost, large‐area neuromorphic vision bionic systems, largely owing to their compatibility with stability, low‐temperature solution processing, and flexible substrates.^[^ ^26^, ^27^, ^28^, ^29^ ^]^ The bidirectional photoresponse in such devices primarily originates from charge transfer effects at the heterojunction interface. In this process, the modulation of electrical conductivity is strongly influenced by the carrier transport behavior at the interface, which depends collectively on factors such as energy level alignment between n‐type and p‐type materials, charge transport properties of individual material components, and the efficiency of interfacial charge transfer.^[^ ^30^, ^31^, ^32^, ^33^ ^]^ Nevertheless, issues such as poor molecular crystallinity, interfacial defects, and interfacial charge accumulation often cause issues such as carrier trapping effects and nonlinear responses. These can lead to photoresponse delays in dynamic lighting scenarios or affect recognition accuracy because of failed feature extraction in low‐contrast images. Furthermore, traditional bidirectional devices often exhibit photoconductive response characteristics insufficiently compatible with neuromorphic computing algorithms. These mismatch limits feature extraction efficiency for low‐contrast images, making consistent recognition accuracy difficult to achieve.

In this study, we demonstrate a robust and effective approach to simulate visual photoadaptation and enable the precise recognition of low‐contrast, non‐ideal images by leveraging the complementary photoconductivity behaviors of two heterojunction structures. We employed the high‐mobility p‐type organic semiconductor 2,8‐difluoro‐5,11‐bis (triethylsilylethynyl) anthradithiophene (diF‐TES‐ADT) as the primary transport layer, strategically combining it with two distinct materials (Dotriacontane (C_32_H_66_), N,N′‐ditridecylperylene‐3,4,9,10‐tetracarboxylic diimide (PTCDI‐C_13_)) to construct dual heterojunction systems: a C_32_H_66_/diF‐TES‐ADT heterojunction exhibiting unidirectional PPC and an n‐p PTCDI‐C_13_/diF‐TES‐ADT heterojunction demonstrating a bidirectional photoresponse dominated by NPC. A comprehensive characterization elucidated the underlying mechanisms governing these photoresponse behaviors, which are critically influenced by heterojunction material selection and interfacial effects. The complementary PPC and NPC behaviors successfully enabled bidirectional simulation of neural signaling, effectively demonstrating the transition from short‐term plasticity (STP) to long‐term plasticity (LTP) to mimic key neurobiological processes. We also developed a mathematical framework to simulate the visual photoadaptation process accurately. More interestingly, building upon the fundamental device characteristics, we optimized the feature extraction performance of convolutional kernels and incorporated an adaptive attention mechanism in a neuromorphic machine vision system. For low‐contrast images under uneven light illumination, the system significantly enhances the edge details and features of the images, and increases the recognition accuracy to 97.4% within ten training cycles, demonstrating remarkable performance advantages in visual information processing in complex lighting environments.

## 2 Results and Discussion

### 2.1 Morphology and Interface Property Analysis of the Heterostructures

**Figure 1:**
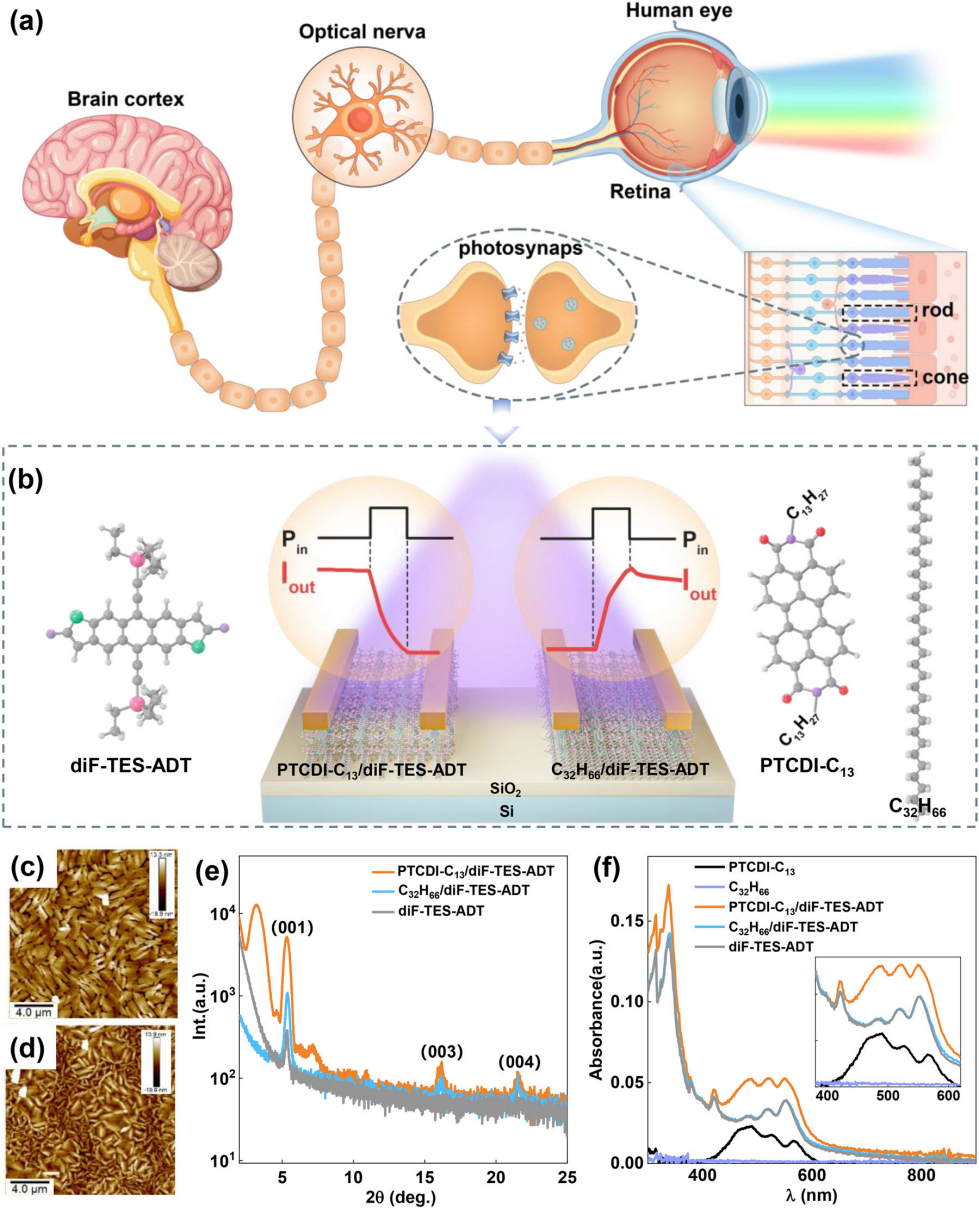
Schematic diagram and interface properties of the heterostructures. a) Schematics of a human eye and a multilayer structure of a retina. b) Device configuration of diF‐TES‐ADT‐based heterostructure phototransistors and the molecular structure of the employed organic materials. c,d) AFM height images of the diF‐TES‐ADT‐based films, (c) PTCDI‐C_13_/diF‐TES‐ADT films, and (d) C_32_H_66_/diF‐TES‐ADT films. e) X‐ray diffraction patterns of the dif‐TES‐ADT‐based films. f) UV–vis–NIR absorption spectra of a series of organic semiconductor films, where the inset shows the amplification in the 400–600 nm range.

The core function of the retina is to perceive light and transmit visual signals to the central nervous system via the optic nerves, where these signals are integrated and processed to form vision. Notably, a more critical functional attribute manifests in robust visual adaptability. This adaptive capacity hinges on the coordinated interplay of diverse cellular populations, with cone and rod cells in the vertebrate retina serving as pivotal mediators: cones perform light detection in bright environments, whereas rods specialize in dim conditions (**Figure** **1**a). Through functional complementarity and dynamic regulation, these two cell types enable adaptation to transition between light and dark environments, ensuring the stable operation of the visual system under varying illumination conditions and exhibiting remarkable adaptive characteristics.^[^ ^17^ ^]^ The light perception and adaptive capability of the retina served as a primary inspiration for our design. Figure 1b shows the configuration of the synapse phototransistors and the molecular structure of the materials. The diF‐TES‐ADT, as a typically high‐mobility and air‐stable p‐type organic semiconductor, is adopted as the transport layer on top.^[^ ^34^, ^35^, ^36^ ^]^ The two different materials used for the bottom layer, N,N′‐ditridecylperylene‐3,4,9,10‐tetracarboxylic diimide (PTCDI‐C_13_) and dotriacontane (C_32_H_66_), present n‐type and neutral transport features, respectively.^[^ ^37^, ^38^ ^]^ On bare SiO_2_ substrates, the diF‐TES‐ADT films display randomly oriented crystals with many grain boundaries and small grain sizes (Figure S1, Supporting Information). However, on the PTCDI‐C_13_ and C_32_H_66_ films (Figure 1c,d), an obvious increase in the crystal domain size but a decrease in the number of grain boundaries are observed in the diF‐TES‐ADT films, which is due to the heteroepitaxial growth effect of flat template layers from the PTCDI‐C_13_ and C_32_H_66_ films (Figure S2, Supporting Information).^[^ ^39^, ^40^ ^]^ In addition, compared with their C_32_H_66_/diF‐TES‐ADT counterparts, PTCDI‐C_13_/diF‐TES‐ADT films are more uniform and larger in size. As shown in Figure S3 (Supporting Information), the C_32_H_66_/diF‐TES‐ADT films exhibit a root‐mean‐square roughness (*R*_q_) of 6.67 nm over a 20 × 20 µm scan. Grain sizes are non‐uniform, ranging from 0.75 ± 0.05 to 1.72 ± 0.1 µm, reflecting variations in the underlying C_32_H_66_ layer. In contrast, the PTCDI‐C_13_/diF‐TES‐ADT films show a lower *R*_q_ value of 5.58 nm over the same scan area and possess an average grain size is 3.21 ± 0.5 µm, which is related to the fact that the growth of diF‐TES‐ADT on C_32_H_66_ depends on the thickness of the template layer. The molecular packing of the diF‐TES‐ADT films on different template layers was further analyzed via X‐ray diffraction (XRD). As shown in Figure 1e, the diF‐TES‐ADT films on both molecular templates show third‐order diffraction peaks that are significantly stronger than those without templates, confirming the high crystallinity of the films. The difference in the template layers leads to a variation in the diffraction peak intensity. The PTCDI‐C_13_/diF‐TES‐ADT film has a stronger diffraction peak intensity, which is consistent with its better crystal morphology. The (001) diffraction peak is located at 2θ = 5.33 ± 0.05°, corresponding to a lattice spacing of 16.57 ± 0.15 Å, which is consistent with the single‐crystal structure of diF‐TES‐ADT.^[^ ^34^, ^41^ ^]^

A series of spectroscopic characterizations were further employed to comprehensively understand the disparities in physical properties exhibited by diF‐TES‐ADT‐based films. As illustrated in Figure 1f, these films demonstrate various absorption degrees within the wavelength (λ) range from 200 to 700 nm, with the most pronounced absorption peak in the ultraviolet (UV) band at ≈365 nm. Notably, when PTCDI‐C_13_ is utilized as the template layer, the photoabsorption ability of diF‐TES‐ADT films is significantly greater than that of those deposited on a bare SiO_2_ substrate or the C_32_H_66_ template. Moreover, substantial intensification is observed in the three absorption peaks between 400 and 600 nm, as depicted in the inset of Figure 1f. This enhancement can be attributed to the inherent photoabsorption ability of the PTCDI‐C_13_ molecule in this specific wavelength range, which increases the overall absorption intensity of the heterostructure PTCDI‐C_13_/diF‐TES‐ADT films. Conversely, for diF‐TES‐ADT films utilizing C_32_H_66_ as the template layer and those deposited on bare SiO_2_ substrates, their absorption curves essentially overlap owing to the negligible photoabsorption ability of C_32_H_66_, thus confirming that the film absorption primarily originates from the diF‐TES‐ADT molecule. The carrier dynamic process of the series of diF‐TES‐ADT‐based films was further characterized by steady‐state photoluminescence (PL) spectra under different excitation wavelengths (λ_ex_), as shown in Figure S4 (Supporting Information). These films demonstrated high fluorescence intensities in the ranges of 375–425 and 550–650 nm, with the C_32_H_66_/diF‐TES‐ADT films exhibiting the highest luminescence intensity among the series. This increase in fluorescence may be attributed to the increased crystallization of C_32_H_66_/diF‐TES‐ADT films on the C_32_H_66_ substrate compared with that on the bare SiO_2_ substrate, along with the absence of a charge transfer response in the C_32_H_66_/diF‐TES‐ADT heterostructure, which results in the strongest luminescence.^[^ ^42^ ^]^ Conversely, the PTCDI‐C_13_/diF‐TES‐ADT films show the lowest luminescence intensity compared with the other films in the series. This pronounced PL quenching suggests that, upon light illumination, charge transfer or exciton dissociation occurs within the heterostructure films.^[^ ^43^, ^44^ ^]^

### 2.2 Photoelectronic Analysis of the Phototransistors

**Figure 2:**
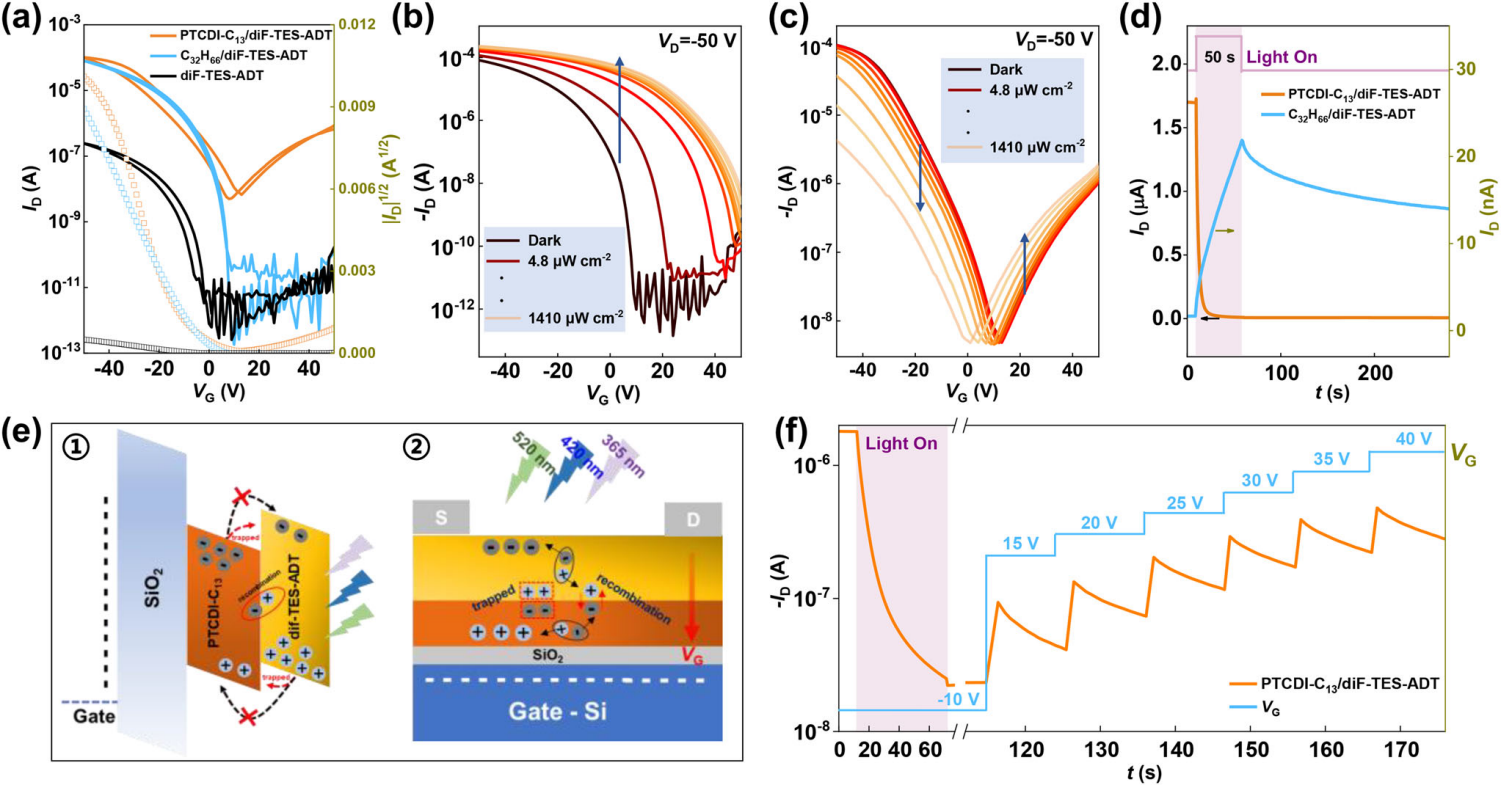
Photoelectronic properties of heterostructure phototransistors. a) Transfer curves (left) and square root of drain current (|*I*_D_|^1/2^) (right) versus gate voltages (*V*_G_) for devices based on diF‐TES‐ADT‐based system phototransistors in the dark (drain‐source voltage (*V*_D_) = ‐50 V). Transfer curves of diF‐TES‐ADT‐based system phototransistors in the dark and under different illumination intensities (λ = 365 nm), b) C_32_H_66_/diF‐TES‐ADT OPTs, c) PTCDI‐C_13_/diF‐TES‐ADT OPTs. d) PPC and NPC transient current response for C_32_H_66_/diF‐TES‐ADT and PTCDI‐C_13_/diF‐TES‐ADT OPTs under UV light irradiation (*P*_in_ = 1.03 mW cm^−2^; *V*_D_ = ‐20 V), where the *V*_G_ of the C_32_H_66_/diF‐TES‐ADT and PTCDI‐C_13_/diF‐TES‐ADT OPTs are 0 V and ‐10 V, respectively. e) Schematic showing the NPC photoresponse mechanism; 1) illustration of the generation, transport, trapping, and recombination processes of photogenerated excitons; 2) energy level diagram of the PTCDI‐C_13_/diF‐TES‐ADT device. f) NPC photoresponse curves under UV illumination and *V*_G_ dual‐modulation.

The electronic properties of the diF‐TES‐ADT‐based photosynapse transistors were tested in advance, and the typical transfer and output characteristics are presented in **Figures** **2**a and S5 (Supporting Information), respectively, and **Table** **1** summarizes the key photoelectrical performance parameters. The two types of diF‐TES‐ADT OFETs, with different molecular templates, generate disparate effects, where PTCDI‐C_13_/diF‐TES‐ADT OFETs exhibit obvious ambipolar transport properties and C_32_H_66_/diF‐TES‐ADT OFETs present typical p‐type transport properties. As demonstrated in Figure S6 (Supporting Information), diF‐TES‐ADT‐based OFETs exhibit good operational stability, maintaining a stable on/off current state with negligible attenuation. Compared with those of single diF‐TES‐ADT OFETs, the mobilities (*μ*) of devices with molecular templates significantly improve, as shown in Figure S7a (Supporting Information), which indicates more efficient charge transport and collection ability. This optimization not only facilitates efficient hole transport pathways within the diF‐TES‐ADT layers but also reduces carrier scattering and the probability of recombination with opposite charges. Moreover, the improved film crystallinity reduces the overall trap density at key interfaces (metal/diF‐TES‐ADT interface and SiO_2_/diF‐TES‐ADT interface), thereby enhancing both charge injection efficiency and transport capability. In addition, the *I*_on_/*I*_off_ ratios of these three devices are shown in Figure S7b (Supporting Information), where C_32_H_66_/diF‐TES‐ADT OFETs have a value of 10^7^, whereas PTCDI‐C_13_/diF‐TES‐ADT has a much lower *I*_on_/*I*_off_ ratio. This should arise from the charge transfer effect at the PTCDI‐C_13_/diF‐TES‐ADT heterointerface or the impact of bipolar transport, resulting in a high channel conductance in the off‐state. Furthermore, the template layers have an effect on the turn‐on voltage (*V*_on_) of the devices, as shown in Figure S7c (Supporting Information). Both PTCDI‐C_13_ and C_32_H_66_ template layers induce a positive shift in the *V*_on_ of the devices. This shift is attributed to the reduction of grain boundaries, which results in fewer carrier trapping sites and consequently facilitates transistor turn‐on.

**Table 1:** The key photoelectrical performance parameters of diF‐TES‐ADT‐based OPTs.

Structure	*μ_h_* [cm^2^ V^−1^⋅s^−1^]	*V*_on_ [V]	*I*_on_/*I*_off_	*R_max_* [AW^−1^]	*P_max_*	*D* ^*^ *_max_* [Jones]
Bare diF‐TES‐ADT	0.0026 ± 0.002	−5.5 ± 2	10^4^	17.2	4.85 × 10^4^	2.46 × 10^13^
PTCDI‐C_13_/diF‐TES‐ADT	1.11 ± 0.2	−13.6 ± 3	10^4^	1.40 × 10^4^	5.73 × 10^6^	1.74 × 10^16^
C_32_H_66_diF‐TES‐ADT	0.41 ± 0.05	−3.4 ± 0.7	10^7^	721.15	0.98	3.7 × 10^12^

The error bar depicts the mean ± SD, *n* = 10

To investigate the photonic synapse behavior of the two heterostructures, the broadband photoresponse characteristics of the OPTs were investigated by using multiple diodes operating from the UV to the visible range (365, 420, 520, 660, and 740 nm), as shown in Figure S8 (Supporting Information). The devices respond obviously to 365, 420, and 520 nm illumination but negligibly respond to 660 and 740 nm light illumination, which agrees well with the absorption spectra of the diF‐TES‐ADT‐based films mentioned previously. Moreover, for C_32_H_66_/diF‐TES‐ADT featuring unipolar electron transport, the current of devices increases with increasing incident light intensity (*P*_in_) irrespective of the wavelength, as shown in Figure 2b and Figure S9 (Supporting Information). The drain current (*I*_D_) under illumination markedly increased as the gate voltage (*V*_G_) increased, suggesting strong amplification of the sensing signals by adjusting *V*_G_ at a fixed source‐drain voltage (*V*_D_). Moreover, compared to bare SiO_2_/diF‐TES‐ADT OPTs, the C_32_H_66_‐modified diF‐TES‐ADT OPTs demonstrate higher photosensitivity (*P*) and responsivity (*R*) (Figure S10, Supporting Information). This is due to the dual role of the C_32_H_66_ modification layer in reducing interface defects and optimizing molecular orientation. This reduction in recombination centers prolongs the lifetime of photogenerated carriers, thereby improving the efficiency of charge separation and collection while suppressing various recombination losses. Collectively, these effects lead to significant enhancement in the *R* and *P* of the C_32_H_66_/diF‐TES‐ADT OPTs. Table S1 (Supporting Information) summarizes the key performance metrics of our devices alongside those of state‐of‐the‐art neuromorphic phototransistors. The comparison, which primarily focuses on neuromorphic phototransistors exhibiting positive photoconductance, demonstrates that our C_32_H_66_/diF‐TES‐ADT OPTs deliver competitive performance.

Interestingly, we found that n‐p PTCDI‐C_13_/diF‐TES‐ADT heterostructure OPTs exhibited completely different characteristics at different *V*_G_ ranges under illuminated conditions, irrespective of the wavelength. As shown in Figure 2c and Figure S9d,e (Supporting Information), the devices exhibited typical PPC characteristics in the electron‐accumulation regime. However, when working in the hole accumulation region, the character of the devices switched from PPC to NPC behavior, where the *I*_D_ decreased abnormally with increasing light intensity. In addition, to further evaluate the two different photoresponse behaviors, the transient responses of n‐p PTCDI‐C_13_/diF‐TES‐ADT and n‐n C_32_H_66_/diF‐TES‐ADT OPTs upon light illumination were recorded, as depicted in Figure 1d. The photocurrent of the C_32_H_66_/diF‐TES‐ADT OPTs starts to increase, whereas that of the PTCDI‐C_13_/diF‐TES‐ADT OPTs decreases under light illumination. After the removal of illumination, the photocurrent is slightly restored and stabilizes at a certain level, indicating the slow decay of the photocurrent and great non‐volatile properties.

**Figure 3:**
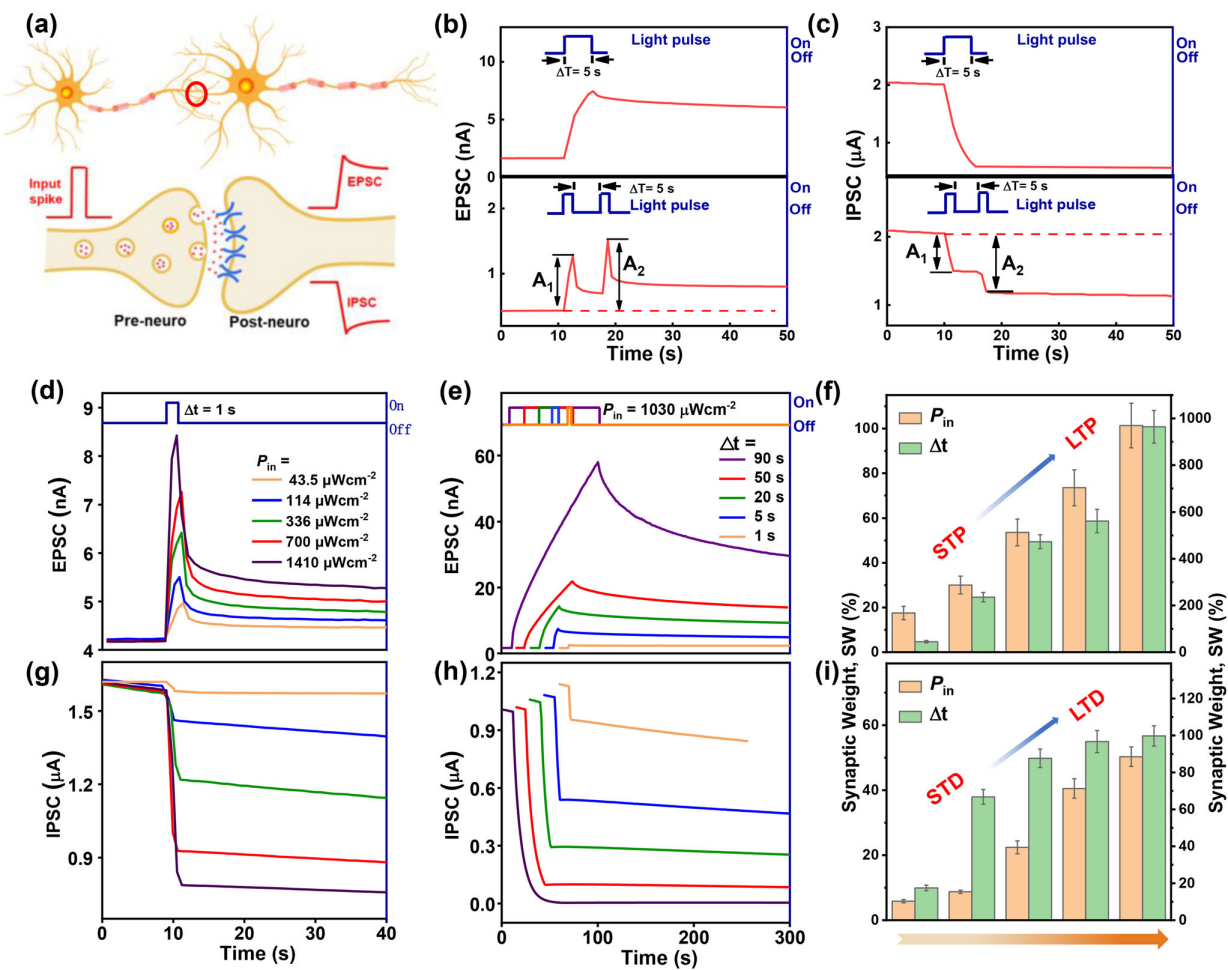
Synapse plasticity of the PPC and NPC phototransistors. a) Schematic diagram of a biological synapse. b) EPSC triggered by a light spike and by two continuous light spikes for C_32_H_66_/diF‐TES‐ADT OPTs (λ = 365 nm; *P* = 1.03 mW cm^−2^), where the light pulse duration for the PPF is 2 s. c) IPSC response stimulated by a single light spike and by two continuous light spikes for PTCDI‐C_13_/diF‐TES‐ADT OPTs (λ = 365 nm; *P* = 1.03 mW cm^−2^), where the light pulse duration for PPD is 2 s. The dependence of the transmission from the STP to LTP on d,g) *P*_in_ and e, h) light pulse width (Δ*t*), d,e) C_32_H_66_/diF‐TES‐ADT OPTs; g,h) PTCDI‐C_13_/diF‐TES‐ADT OPTs (*V*_G_ = ‐10 V; *V*_D_ = ‐20 V). The changes in SW were measured for the diF‐TES‐ADT‐based synapse phototransistors by varying the *P*_in_ and Δ*t*, (f) C_32_H_66_/diF‐TES‐ADT OPTs; (i) PTCDI‐C_13_/diF‐TES‐ADT OPTs (the error bar depicts the mean ± SD, *n* = 5).

According to our previous investigation, the direction of the gate electric field and the direction of exciton dissociation greatly contribute to the negative photoresponse behavior.^[^ ^45^ ^]^ To explain the charge carrier transfer and NPC phenomenon, the device schematics and band structures of the n‐p heterojunction PTCDI‐C_13_/diF‐TES‐ADT OPTs before and after applying light pulses and different *V*_G_ values are compiled in **Figure** **3**e and Figures S11 and S12 (Supporting Information). First, it should be emphasized that in the absence of external bias and solely under the influence of the built‐in electric field, the charge separation or exciton dissociation process in organic p‐n heterojunction semiconductors follows a natural mechanism, where electrons tend to transfer toward the acceptor layers while holes remain in the donor layers.^[^ ^46^ ^]^ Therefore, in the PTCDI‐C_13_/diF‐TES‐ADT n‐p interface with similar energy level structures, when there is no *V*_G_, the spontaneous dissociation of excitons and the separation of charges are that photogenerated holes preferentially remain in the diF‐TES‐ADT layer and that photogenerated electrons transfer to the PTCDI‐C_13_ layer. However, when *V*_G_ is applied, an electric field is generated at the interface, which significantly affects the dissociation behavior of excitons and the subsequent charge separation process.

Specifically, under a positive *V*_G,_ as shown in Figure S11a (Supporting Information), the electric field points from PTCDI‐C_13_ to diF‐TES‐ADT, causing electrons to transfer toward the interface or the PTCDI‐C_13_ layers, whereas holes remain and accumulate in the diF‐TES‐ADT layer. This charge distribution state facilitates the efficient transport of both types of carriers and contributes positively to the current of the conductive channel. Upon illumination, the photogenerated holes and electrons follow the aforementioned paths, thereby increasing the hole current and exhibiting a positive photoconductive effect (Figure S11b,c, Supporting Information). Conversely, under negative *V*_G_, the electric field direction is reversed, pointing from diF‐TES‐ADT to PTCDI‐C_13_. This change will cause electrons to aggregate toward the diF‐TES‐ADT layer, whereas holes will migrate toward the PTCDI‐C_13_ layer. Then, electrons and holes at the interface may undergo recombination or become trapped, which has no positive effect on the conductivity of the channel (Figure S12a, Supporting Information). After illumination, the photogene*r*ated holes and electrons continue to follow the aforementioned migration paths: photoelectrons in the PTCDI‐C_13_ layers are driven by the electric field to the upper diF‐TES‐ADT layers, where they may recombine with holes from the diF‐TES‐ADT layers or be trapped at the interface; simultaneously, photogenerated holes in the diF‐TES‐ADT layer are pushed by the electric field to the lower PTCDI‐C_13_ layers (Figure S12b, Supporting Information). This charge distribution results in a decrease in the hole density of the diF‐TES‐ADT layers. The photogenerated holes in the PTCDI‐C_13_ layer are driven by the electric field to the SiO_2_ surface and captured. The key evidence for this process is the negative shift in the threshold voltage (*V*_TH_) observed in the transfer characteristics of the PTCDI‐C_13_/diF‐TES‐ADT OPTs under increasing illumination intensity (Figure S13, Supporting Information). This *V*_TH_ shift signifies that a progressively larger gate voltage is required to form a hole accumulation channel. This phenomenon is a classic signature of the photogating effect. Under negative *V*_G_, the electric field drives photogenerated holes from the PTCDI‐C_13_ layer toward the dielectric interface. The subsequent trapping of these holes at the SiO_2_ surface creates a localized positive space charge. This positive charge electrostatically counteracts the applied negative gate voltage, effectively shielding the channel current from the gate field. Consequently, a more negative *V*_G_ is needed to achieve the same level of hole accumulation, which is directly measured as the observed negative *V*_TH_ shift. This phenomenon may induce a photogate effect that reduces the gate bias, making it more difficult for holes to accumulate in the channel, thereby requiring a higher threshold voltage to form an effective channel.

To verify the aforementioned speculation, we adjusted the magnitude and direction of the *V*_G_ electric field during the transient response tests of the PTCDI‐C_13_/diF‐TES‐ADT OPTs. As illustrated in Figure 2f, initially, under the application of a negative *V*_G_ (*V*_G_ = ‐10 V), the device exhibited a significant decrease in the photocurrent upon illumination, whereas after illumination was removed, the current remained stable without significant attenuation. Subsequently, we reversed the direction of the *V*_G_ electronic field and gradually increased the value, observing a continuous increase in the device current. This phenomenon clearly indicates that reversing the direction of the electric field facilitates the redistribution of electrons and holes within the PTCDI‐C_13_ and diF‐TES‐ADT layers, thereby reducing the potential recombination rate and trapping effect at the interface and effectively increasing the number of electrons in the PTCDI‐C_13_ layers and the holes in the diF‐TES‐ADT layers. In addition, we observed a slow decay trend in the current when a positive *V*_G_ was applied, which might be triggered by switching the *V*_G_ from negative to positive. The entire process is governed by the redistribution of charge carriers, including re‐trapping and recombination of charges. Initially, under the prior negative *V*_G_, the gate‐induced electric field was directed from the diF‐TES‐ADT layer toward the PTCDI‐C_13_ layer. The photogenerated holes in the PTCDI‐C_13_ layer were driven toward the SiO_2_ interface and became trapped, thereby inducing a photogating effect that screened the applied gate field. Concurrently, the photogenerated electrons in the PTCDI‐C_13_ layer were driven by the gate field into the upper diF‐TES‐ADT layer, where they could recombine with holes from diF‐TES‐ADT or become trapped at the heterointerface (Figure S14a, Supporting Information). These processes resulted in a decrease in hole concentration within the diF‐TES‐ADT channel, leading to a reduction in channel conductivity and manifesting as negative photoconductivity (NPC). Subsequently, when the *V*_G_ is switched from negative to positive, the direction of the electric field across the PTCDI‐C_13_/diF‐TES‐ADT heterostructure reverses, now pointing from PTCDI‐C_13_ toward diF‐TES‐ADT. This reversal causes electrons to be pulled back into the PTCDI‐C_13_ layer close to the SiO_2_ interface, while holes are accumulated and retained in the diF‐TES‐ADT layer, gradually dissipating the prior photogating effect (Figure S14b, Supporting Information). The recombination and trapping phenomena at the heterointerface are significantly suppressed. Consequently, the charge carrier concentration in the channel – specifically, holes in the diF‐TES‐ADT layer and electrons in the PTCDI‐C_13_ layer – increases rapidly, causing an instantaneous increase in the drain current. This rapid increase is transient, as the redistribution of carriers occurs promptly in response to the altered electric field. However, following this rapid current increase, a slow decay is observed. The trap states or charge capture centers still persist at the PTCDI‐C_13_/diF‐TES‐ADT interface or SiO_2_ interface. Specifically, at the SiO_2_ interface, the holes trapped during the previous negative *V*_G_ now recombine with electrons originating from the channel or the n‐type layer, which gradually eliminates the photogating effect and reduces the effective gate voltage, contributing to current slightly decay (Figure S14c, Supporting Information). Simultaneously, at the PTCDI‐C_13_/diF‐TES‐ADT heterointerface, electrons and holes reach a new steady‐state concentration through recombination and trapping dynamics. Therefore, the current slowly decreases from its peak value. This slow decay is a characteristic relaxation behavior in organic heterostructures, analogous to persistent photoconductivity phenomena.

### 2.3 Photonic‐Synapse Characteristics of the PPC and NPC Phototransistors

In biological synapses, neurotransmitters are released from presynaptic neurons upon receiving external stimulus signals, and the postsynaptic potential varies with neurotransmitter stimulation, triggering excitatory and/or inhibitory postsynaptic potentials and eventually generating excitatory postsynaptic currents (EPSCs) or inhibitory postsynaptic currents (IPSCs) across synapses.^[^ ^47^, ^48^, ^49^ ^]^ Here, the stable and controllable persistent photocurrent decay behavior observed in the diF‐TES‐ADT‐based heterostructure OPTs offers potential possibilities for the simulation applications of artificial visual systems. Figure 3a presents a schematic of the biological synapse. Herein, the stimulation of the UV light (365 nm) pulse was utilized as an action potential that acted on the pre‐neurons, and the *I*_D_ was defined as the postsynaptic current (PSC), which is frequently utilized as an output signal to simulate the synaptic connection strength between neurons. As depicted in Figure 3b, upon exposure to light stimulation, the PSC value of the C_32_H_66_/diF‐TES‐ADT synaptic phototransistor increases sharply, indicating a significant EPSC. When the ultraviolet light is switched off, the PSC values decay slowly, eventually maintaining a relatively high level compared with the initial current value. In contrast, for the PTCDI‐C_13_/diF‐TES‐ADT synaptic phototransistor, a sharp decrease in the PSC is observed, indicating prominent IPSC characteristics. Notably, the current failed to recover after the light pulse was turned off, which might be attributed to the slow release of trapped holes and subsequent delayed recombination of trapped electrons and holes, thereby resulting in extremely slow decay times and prolonged relaxation periods. Moreover, at a shorter light pulse switch (Δ*t* = 0.3), C_32_H_66_/diF‐TES‐ADT and PTCDI‐C_13_/diF‐TES‐ADT synaptic phototransistors still maintained excellent excitatory and inhibitory synaptic behavior (Figure S15, Supporting Information). The bidirectional PSC responses of EPSCs and IPSCs play a mutually complementary role in the information transmission of neural networks, which is essential for the normal operation of neural communication. Paired‐pulse facilitation (PPF)/paired‐pulse depression (PPD), a notable manifestation of short‐term plasticity prevalent in both excitatory and inhibitory synapses, can be employed as a means to assess the performance of diF‐TES‐ADT‐based synaptic devices.^[^ ^50^, ^51^ ^]^ This refers to the phenomenon of enhanced postsynaptic responses induced by two consecutive presynaptic pulses. This behavior is highly important in the recognition and decoding of information in a biological neural system. In Figure 3b,c, the lower side images illustrate the measurements of EPSCs or IPSCs induced by a pair of presynaptic spikes. Notably, the maximum amplitude of the EPSC (IPSC) generated by the second presynaptic spike is significantly larger than that of the first presynaptic spike, which is consistent with the typical PPF (PPD) behavior of biological synapses.

Short‐term potentiation (STP), long‐term potentiation (LTP), short‐term depression (STD), and long‐term depression (LTD) are pivotal synaptic plasticity mechanisms in the hippocampus of the brain and are essential for learning and memory processes.^[^ ^52^, ^53^, ^54^, ^55^ ^]^ They not only facilitate the creation and consolidation of new memories but also contribute to the refinement, updating, and correction of existing memories. For example, LTP enhances memory formation, whereas LTD facilitates the deletion or resetting of these memory traces in the brain, thereby creating space for the storage of new information. In addition, synaptic plasticity refers to the dynamic regulatory ability of synapses to adjust the transmission efficiency between pre‐ and postsynaptic neurons, quantified by the synaptic weight (SW, SW = |(PSC_sw_−PSC_in_)/PSC_in_|, Figure S16, Supporting Information). Here, by manipulating the power density and width of light pulses, the intensity of learning can be mimicked, with the measured *I*_D_ employed as an indicator of the memory level. Through the reinforcement training process, photonic synaptic behavior increases in SW, transitioning from a weak to a strong state and shifting from short‐term plasticity to long‐term plasticity. For the C_32_H_66_/diF‐TES‐ADT synaptic phototransistor shown in Figure 3d,e, with increasing learning intensity, the peak photocurrent gradually increases to higher values. Moreover, the decay of the photocurrent indicates typical memory behavior, and the memory retention time increases with increasing light pulse intensity and width, which is highly similar to the learning and forgetting behavior of the human brain. Additionally, with increasing learning intensity, represented by greater power density and broader light spike width, the decay rate of the memory level slows, indicating a transition from STP to LTP (Figure 3f). Conversely, for the PTCDI‐C13/diF‐TES‐ADT synaptic transistor, an increase in the light pulse intensity and width results in a gradual reduction in current, indicating that each pulse causes inhibition (Figure 3g,h). The synaptic weight accumulates and gradually decreases with increasing number of applied pulses, mimicking the typical LTD phenomenon. Furthermore, with an increasing number of pulses or pulse width, a greater degree of depression is observed, facilitating the transition from STD to LTD, as illustrated in Figure 3i. In addition, by manipulating the number of light pulses, the intensity of learning can be mimicked, with the measured *I*_D_ employed as an indicator of the memory level (Figure S17, Supporting Information). Through the reinforcement training process, photonic synaptic behavior increases in SW, transitioning from a weak to a strong state and shifting from short‐term plasticity (STP) to long‐term plasticity (LTP), which is highly similar to the learning and forgetting behavior of the human brain. Moreover, the characteristic time constant of C_32_H_66_/diF‐TES‐ADT synaptic phototransistor, derived from exponential fits to the decay curves (*y = Aexp(‐x/t)+B*), showed a progressive increase with the number of light pulses, suggesting a retardation of the current decay process. This behavior exhibits a trend analogous to the learning and forgetting mechanisms in biological systems. It should be noted that the decay curves of PTCDI‐C_13_/diF‐TES‐ADT OPTs could not be fitted, due to current of PTCDI‐C_13_/diF‐TES‐ADT OPTs does not decay after removal of light. By precisely simulating the STP, LTP, STD, and LTD, artificial synapse transistors can achieve full‐process simulation from short‐term memory to long‐term memory, thereby providing robust theoretical and technological foundations for the development of more efficient and intelligent artificial neural networks.

In addition, the stability tests on the diF‐TES‐ADT‐based synaptic phototransistors by subjecting them to 60 consecutive optical pulse cycles. As shown in Figure S18 (Supporting Information), C_32_H_66_/diF‐TES‐ADT OPTs and PTCDI‐C_13_/diF‐TES‐ADT synaptic phototransistors exhibited excellent operational stability throughout the cyclic illumination. A significant non‐volatile memory characteristic was consistently observed after the light was removed, underscoring their reliability for practical applications. However, for NPC PTCDI‐C_13_/diF‐TES‐ADT OPTs, when the current drops to a certain critical value, their behavior will transition to positive photoswitching behavior, although the overall current still shows a downward trend (Figure S19, Supporting Information). This transformation is mainly due to the insufficient number of holes in the diF‐TES‐ADT layers available for sustained recombination when the hole concentration is depleted to a certain extent. At this point, although the illumination continues, the generation and recombination process of charge carriers reaches a new steady state, exhibiting positive photoresponse properties. Then, long‐term current decay tests on C_32_H_66_/diF‐TES‐ADT OPTs and PTCDI‐C_13_/diF‐TES‐ADT synaptic phototransistors after removing light exposure as shown in Figure S20 (Supporting Information), and it can be seen that the device still maintains a high current level after 30 min of light removal, demonstrating excellent non‐volatile performance. Furthermore, about the multi‐level state of diF‐TES‐ADT‐based synaptic phototransistors as shown in Figure S21 (Supporting Information), the device conductance can be progressively and stably switched to ≈20 discrete and distinguishable states as the number of pulses increases. Each conductance state exhibits excellent non‐volatile retention characteristics after every pulse along with quasi‐linear overall trend in the conductance increase with the number of pulses. These 20 stable and distinct states indicate that, under our experimental conditions, the device can achieve a weight precision of log_2_(20) ≈4.3 bits. This level of precision meets the fundamental requirements for synaptic devices in neuromorphic computing, which typically demand several tens of stable states.

### 2.4 Mimicking Human Visual Photoadaptation Functionality

**Figure 4:**
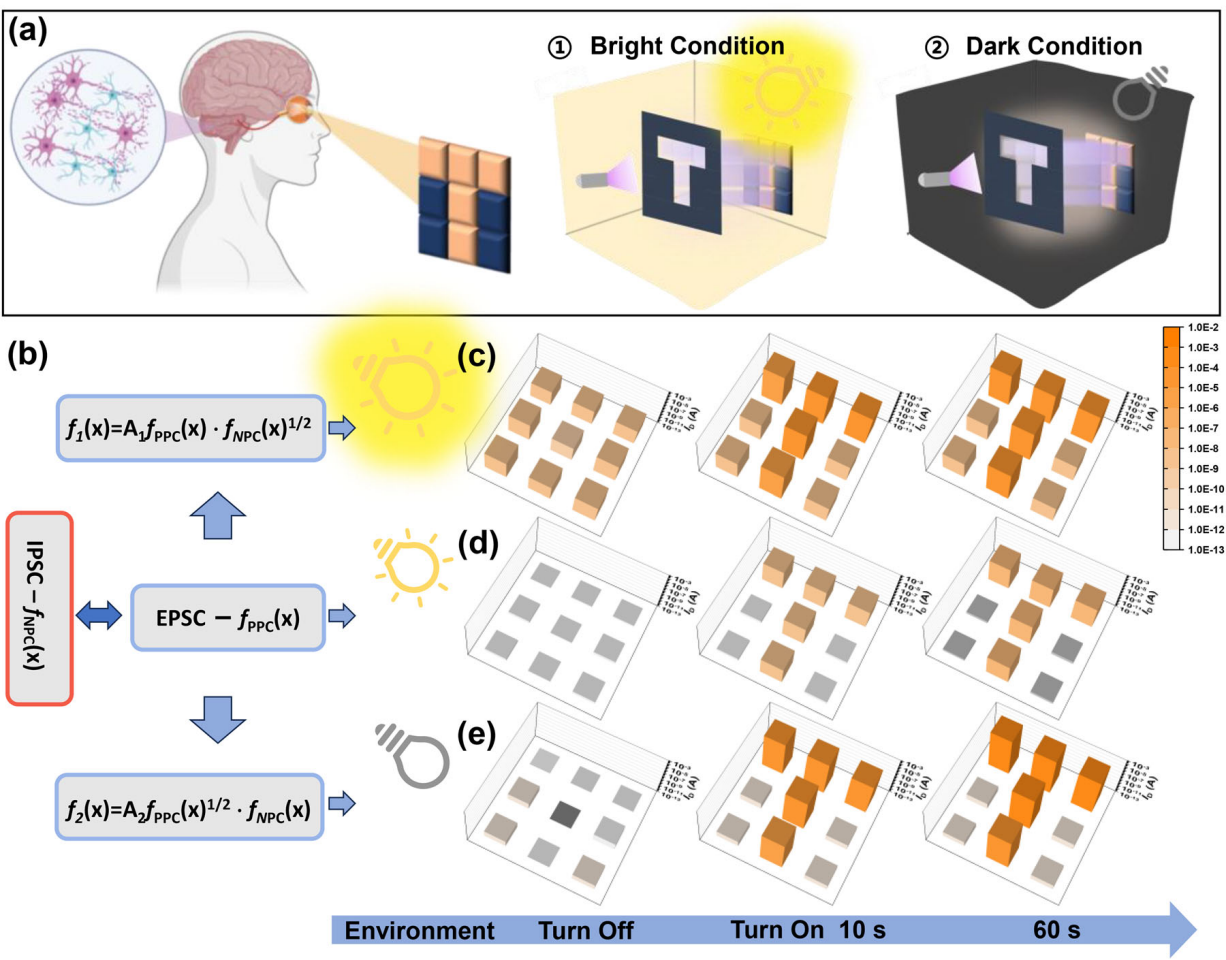
Simulation of human visual photoadaptation. a) Schematic representation of the human visual system and a 3 × 3 pixel array under bright and dark conditions. b) Mathematical fitting function of the photocurrent value based on time variation, where the photocurrent function based on the PPC effect (C_32_H_66_/diF‐TES‐ADT OPTs) is *f*_PPC_(x) and the photocurrent function based on the NPC effect (PTCDI‐C_13_/diF‐TES‐ADT OPTs) is *f*_NPC_(x). A_1_ and A_2_ are constants whose values are 10^7^ and 10^19^, respectively. c–e) Simulation of the adaptation process of human vision to a T image. (c) Under bright conditions; (d) under normal conditions; (e) under dark conditions.

Human visual perception involves a sophisticated adaptive mechanism that enables us to maintain clarity and contrast in visual sensing under varying lighting conditions.^[^ ^56^ ^]^ This adaptability involves both rapid responses to changes in brightness and long‐term adjustments to different light environments, ensuring effective perception and understanding of the surroundings under diverse illumination conditions.^[^ ^21^, ^57^, ^58^ ^]^ Dynamic visual adaptation to varying light stimuli is a key capability of photonic synaptic devices. To simulate the visual adaptation activities of human eyes under different environmental lighting conditions, we designed a 3 × 3 phototransistor array to sense the patterns of the letter “T” at a fixed UV light intensity of 1.03 mW cm^−2^. Even when the light illuminating the array remains constant, the perceived illuminance by the human eye changes in response to variations in the surrounding ambient light, demonstrating automatic visual adaptation to different lighting conditions, as shown in **Figure** **4**a. Drawing inspiration from this biological phenomenon, we introduce simple mathematical functions into the PPC and NPC photoresponse curves of the array, as depicted in Figure 4b, successfully constructing an artificial visual neural system that integrates perception, storage, and computation, thereby effectively mimicking the adaptive characteristics in human vision. Under standard illumination conditions, upon the activation of UV light, the device array precisely exhibits the prestimulus initial state, the poststimulus activated state, and the progressive adaptation process during the stimulation period (Figure 4d). This manifestation is highly congruent with the adaptation phenomenon detected by human vision. Additionally, the recognition response of the device array to the letter “T” gradually increases from the initial low level to a stable adaptive state. This not only demonstrates the dynamic visual adaptability of the photonic synapse transistor but also validates its effectiveness in simulating human visual adaptation.

Under strongly bright environmental light conditions (Figure 4c), when illuminated by the same UV light, regions of the T‐shaped array with a larger current show saturation and overexposure phenomena, temporarily affecting the precise identification of spatial resolution. Moreover, as the light exposure continues, the “T” pattern gradually becomes clearer within a short time, indicating that our devices possess great active light adaptation capabilities. Conversely, under dark background environmental conditions (Figure 4e), when illuminated by the same UV light, the current distribution of the device array becomes remarkably clear, presenting a high‐contrast T‐shaped image. These phenomena not only verify the adaptability of photonic synapse transistors to different environmental light conditions but also demonstrate their exceptional image recognition and presentation performance in low‐light conditions. Therefore, by modulating the device photoresponse behavior and introducing simple algorithmic processing, the photonic synaptic transistor successfully emulates the adaptive characteristics of human vision and has significant potential for broad applications in artificial visual neural systems that integrate perception, storage, and computation.

### 2.5 Simulation of Machine Vision Systems

**Figure 5:**
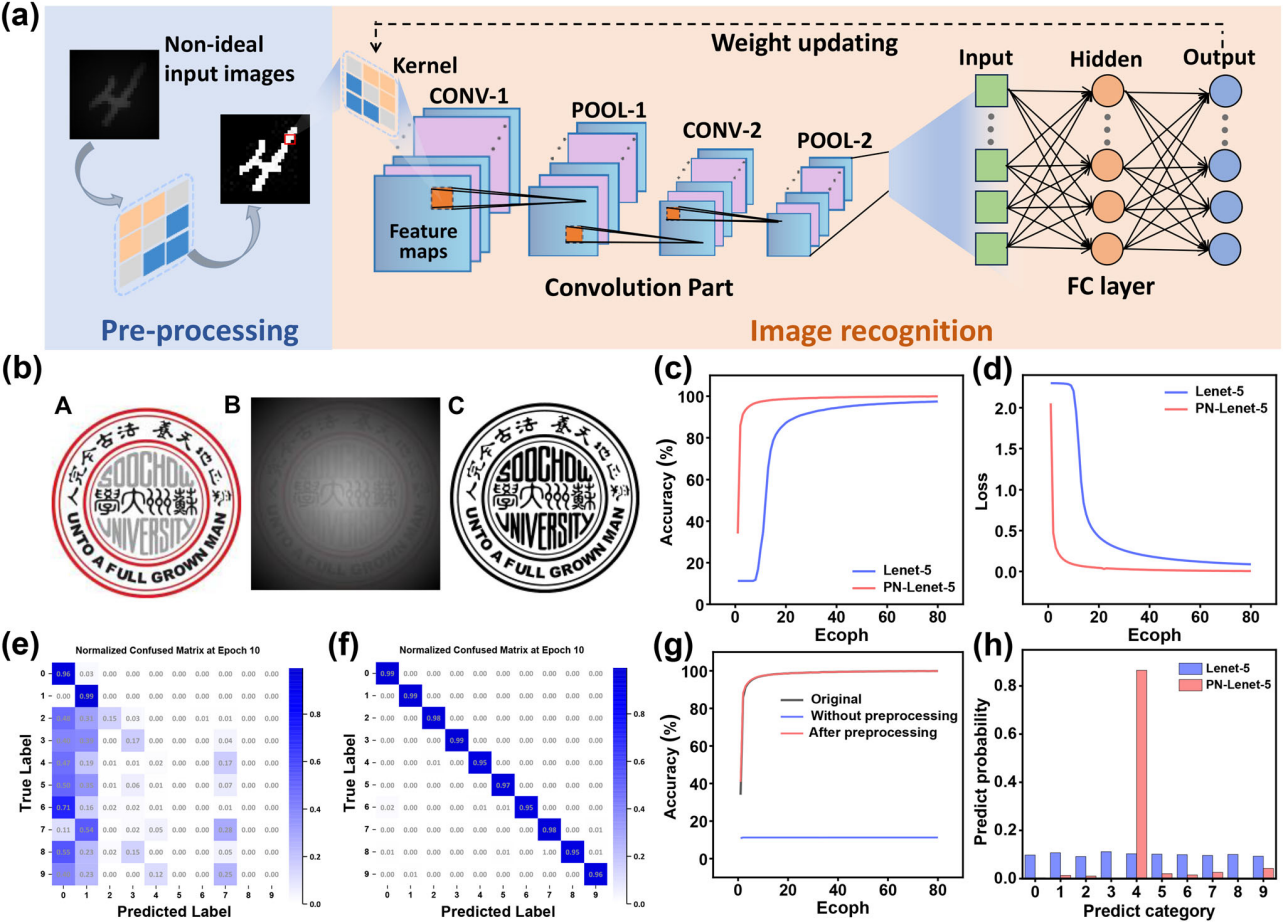
Simulation of machine vision systems combined with PPC and NPC effects. a) Schematic diagram depicting the preprocessing and image recognition procedures of PN‐Lenet‐5 architectures for low‐contrast images, which involve contrast enhancement, feature extraction, and classification. b) The emblem of Soochow University is presented as follows: A) the original image; B) the non‐ideal input image under uneven illumination and low contrast ambient conditions; and C) the ideal input image that has been preprocessed. c) Recognition accuracy and d) loss function for CNNs with different architectures. e,f) Normalized confusion matrix at 10 epochs. (e) Lenet‐5; (f) PN‐Lenet‐5. g) Recognition accuracy of PN‐lenet‐5 for the low‐contrast MNIST dataset with and without preprocessing. h) Comparison of the prediction probabilities of the digits of the MNIST dataset at 10 epochs for PN‐Lenet‐5 and Lenet‐5.

Machine vision faces complex and variable lighting conditions, commonly including low‐light environments, interference from multiple light sources, and partial occlusion of light. Images captured under such adverse conditions generally suffer from significant contrast deficiencies. This low‐contrast phenomenon blurs the contours of the detection targets and causes a loss of detail. As a result, it becomes difficult to extract key feature information effectively from images, such as edges, textures, and shapes. The absence of this crucial feature information not only reduces the recognizability of the images but also poses a substantial challenge to subsequent image processing and analysis tasks. Therefore, how to accurately extract and enhance image features under adverse lighting conditions has emerged as one of the key issues that urgently needs to be solved in the field of machine vision. Currently, although a small number of research efforts have indicated that it is possible to develop machine vision systems for image feature recognition and classification on the basis of positive and negative photoconductive effects, relevant research remains rather limited.^[^ ^7^, ^59^, ^60^ ^]^ In light of the current research status and practical requirements, a machine vision system using organic heterojunction transistors with positive and negative photoconductive effects was constructed, and **Figure** **5**a presents a schematic diagram of the entire process.

During the image preprocessing stage, the positive and negative photoconductive response enhancement behavior, which is based on light intensity input, can perform sliding threshold binarization on local pixel clusters, thereby significantly enhancing the sharpness of edges and overall contrast. This technique uses the gray‐level values of the image, corresponding to light intensity, as input and dynamically adjusts weights within a local range to adapt to the average characteristics of different pixel clusters. Specifically, pixels above the threshold are enhanced, whereas those below the threshold are suppressed. This process effectively enhances details and highlights features in images captured under complex conditions. To intuitively demonstrate the effectiveness of this technique, we used the emblem of Soochow University (Figure 5b‐A) as an example to simulate the processing of the algorithm (details illustrated in Note S1, Supporting Information). After preprocessing, as shown in Figure 5b‐B and Figure 5b‐C, the originally blurred image, caused by uneven light illumination and low contrast, presented significantly enhanced edge details and features. In contrast, traditional image processing algorithms, which cannot dynamically adjust parameters, often result in insufficient feature detail extraction and noise enhancement, as depicted in Figure S22 (Supporting Information). Accurate detection of low‐contrast sub‐images can provide more targeted information for subsequent image processing. This enables the application of appropriate enhancement algorithms in later stages to improve image quality, thereby increasing the accuracy and reliability of the entire image recognition system.

In the subsequent image recognition section, we constructed two convolutional neural network (CNN) architectures, which are the classic Lenet‐5 network and its improved version, and the positive‐negative photoconductive response is enhanced by Lenet‐5 (PN‐Lenet‐5). In PN‐Lenet‐5, we introduce a convolution kernel based on the unique positive‐negative photoconductive properties of the aforementioned devices for feature extraction (details illustrated in Note S2, Supporting Information). Additionally, we incorporated the attention mechanism into the architecture of PN‐Lenet‐5, forming an adaptive attention enhancement mechanism based on positive‐negative photoconductive characteristics (PN‐Attention Enhancement) (details illustrated in Note S3, Supporting Information). The core concept of this mechanism is that when processing information, the model does not need to focus on all the information. Instead, it weighs the most important features according to the task requirements. In PN‐Lenet‐5, PN‐Attention Enhancement enables the model to concentrate on the most crucial features by calculating and weighing the importance of each region. The output is subsequently processed through convolutional and pooling layers. The high‐dimensional features are flattened via the fully connected layer, and the SoftMax function is used to output the recognition probability of each category. During the back‐propagation process of the image recognition task, the attention weights and relative importance of positive‐negative photoconduction are dynamically updated and adjusted on the basis of historical results, thereby continuously improving the performance of the PN‐Lenet‐5 model in subsequent training epochs.

To validate the superiority of PN‐Lenet‐5 in preprocessing and subsequent training, we used the National Institute of Standards and Technology (MNIST) dataset for training and recognition of the CNN. In the model evaluation process, we employed multiple metrics, including accuracy, loss value, and F1 score. Accuracy measures the ratio of the number of correctly predicted samples to the total number of samples by the model. The loss value reflects the difference between the predicted results of the model and the actual values. The F1 score, which is the harmonic means of precision and recall, comprehensively considers the precision and completeness of the model, especially in cases of class imbalance, and has an important reference value. Moreover, we conducted a visual analysis of the model's prediction results via a confusion matrix to identify which categories the model is prone to misclassification and which categories have higher prediction accuracy. As shown in Figure 5c, the PN‐Lenet‐5 model increased the training accuracy to over 90% within 5 epochs, whereas the classic Lenet‐5 model required five times as long to achieve the same training effect. Similarly, the comparison of the loss functions of the two models in Figure 5d also demonstrates the advantage of PN‐Lenet‐5 in terms of convergence speed. The confusion matrices of the models at 10 epochs in Figure 5e,f indicate that PN‐Lenet‐5 can perform the recognition task well within 10 epochs. After training saturation, the F1 score of PN‐Lenet‐5 is 2% higher than that of the classic Lenet‐5 model (Figure S23, Supporting Information). The main reason for this performance improvement is the integration of the adaptive attention enhancement features of positive‐negative photoconduction shown in the above heterostructure OPTs, which can quickly filter out redundant information and precisely locate key features. With this feature, the PN‐Lenet‐5 system has significant advantages in complex scenarios, such as suspect facial feature recognition in intelligent security and product quality inspection on industrial automation production lines.

Finally, to demonstrate the application potential of the PN‐Lenet‐5 machine vision system in simulating artificial visual processing, the low‐contrast MNIST dataset that exceeds the human eye resolution limit was preprocessed and input into PN‐Lenet‐5 for recognition. As shown in Figure 5g, the recognition accuracy of the preprocessed dataset is slightly better than that of the original MNIST dataset. However, for low‐contrast images without preprocessing, the model is almost unable to accurately recognize features, with the recognition accuracy limited to ≈11%. As a demonstration, the recognition of the MNIST dataset of digit “4” is shown in Figure S24 (Supporting Information). For better visualization, we selected the classic Lenet‐5 and PN‐Lenet‐5 architectures trained for 10 epochs for comparison (Figure 5h). For PN‐Lenet‐5, the probability of predicting digit “4” is the highest, and the prediction is the most accurate; in contrast, for Lenet‐5, the prediction probabilities for each digit are quite evenly distributed, indicating that it fails to make an effective prediction. In summary, by combining the excellent characteristics of the positive‐negative photoconductive effect, the entire image recognition system has advantages over classic computer vision. The PN‐Lenet‐5 architecture can achieve image recognition in a high‐dynamic range and faster optimization, indicating its potential applications in real‐time machine vision systems, such as intelligent glasses for blind people to recognize traffic lights and other obstacles, and real‐time industrial monitoring for high‐temperature or low‐contrast defect detection.

## 3 Conclusion

In summary, we have reported a simple yet effective approach for simulating visual photoadaptation and precisely recognizing low‐contrast, non‐ideal images by leveraging the complementary photoconductivity of two heterojunction structures. Owing to differences in interface charge transfer and transport characteristics, two highly ordered organic heterojunctions exhibit NPC and PPC synaptic behavior, serving as a primary inspiration for the design. Complementary photonic synapse behaviors enable bidirectional neural signaling simulation and mimic neurobiological processes. By integrating these characteristics into CNN and simultaneously optimizing algorithm architecture, the details and edges of low‐contrast images are markedly enhanced. The system can increase the accuracy of image recognition to 97.4% within ten cycles. This work offers a novel idea to construct the bidirectional photoadaptive synaptic transistors for the development of high‐performance neuromorphic machine visual systems, making them promising candidates for in‐sensor computing applications.

## 4 Experimental Section

### Materials and characterization

The substrate material was a highly doped n‐type silicon (Si) wafer that was used as a gate electrode with a resistivity of less than 0.005 Ω cm and a 300 nm thermally deposited oxidized silicon (SiO_2_) layer whose dielectric capacitance was *C*_i_ = 10 nF cm^−2^ as a dielectric layer. The samples were ultrasonically cleaned in acetone, ethanol, and deionized water for 20 min and then dried with a nitrogen gun. The organic small molecule Dotriacontane (C_32_H_66_) was purchased from Alfa Aesar Company. N, N′‐ditridecylperylene‐3,4,9,10‐tetracarboxylic diimide (PTCDI‐C_13_) and 2,8‐difluoro‐5,11‐bis(triethylsilylethynyl) anthradithiophene (diF‐TES‐ADT) materials were purchased from Lumtec Company. All the materials were used as received without further purification. All the films were prepared via vacuum deposition with a vacuum < 10^−4^ Pa, where the substrate temperature of PTCDI‐C_13_ was 200 °C and the other materials were heated at room temperature at a growth rate 1–2 nm min^−1^. The thickness, measured in situ with a high‐precision quartz crystal microbalance (QCM) monitor (SI‐TM508/608), are 35 nm for diF‐TES‐ADT and 5 nm for both C_32_H_66_ and PTCDI‐C_13_. The morphology of the films was characterized via atomic force microscopy (AFM, Bruker Dimensional Icon) in tapping mode. X‐ray diffraction (XRD, Bruker D8 Discover) in out‐of‐plane reflection mode (λ = 1.54056 Å) was used to characterize the crystallization quality of the films. The operating voltage was 40 kV, and the operating current was 40 mA. UV–vis–NIR absorption spectra were obtained on a Perkin Elmer Lambda 950 spectrophotometer, where the quartz was used as the substrate.

### Device Fabrication and Measurements

40 nm gold was thermally evaporated through a shadow mask whose channel length (*L*) and width (*W*) were 50 and 1000 µm, respectively, and the deposition rate was controlled at 0.3 Å s^−1^. The performance of the organic phototransistors was measured via a Keithley 4200 SCS semiconductor parametric analyzer integrated with a high‐vacuum probe station (Lake Shore) at room temperature. The saturation mobility can be estimated via the equation $\mu =\frac{2L}{WC_{i}}\;{\left(\frac{\sqrt[{\partial }]{I_{D}}}{\partial V_{G}}\right)}^{2}$, where *W* and *L* are the channel width and length, respectively, and where *C_i_* is the capacitance of the SiO_2_ dielectric unit area (*C_i_* ═ 10 nF cm^−2^). Monochromatic light‐emitting diodes (LEDs) with various wavelength sources, driven by a commercial illuminant (CEAULIGHT CEL‐LEDS35), were employed as light sources to illuminate the conductive channels, and the incident direction of light was perpendicular to the device plane. The light power density was calibrated via an optical power meter (Thorlabs Inc., Model PM400). The responsiveness (*R*), sensitivity (*P*), and detection rate (*D* ^*^) of the phototransistors are given by the following formulas: *R* = (*I*_ill_‐*I*_dark_)/(*P*_inc_ *A*), *P* = (*I*_ill_–*I*_dark_)/*I*_dark_, *D* = *A* ^1/2^ *R*/(2*qI*_dark_)^1/2^, where *I*_dark_ and *I*_ill_ are the drain currents in the dark and under illumination, respectively. *P*_inc_ is the incident light power density, and *A* is the area of the illuminating channel.

## Conflict of Interest

The authors declare no conflict of interest.

## Supporting information



Supporting Information

## Data Availability

The data that support the findings of this study are available from the corresponding author upon reasonable request.
