# Supplementary material for: Bidirectional Photoadaptive Organic Heterojunction Synaptic Transistors for Accurate Image Recognition in Machine Vision Systems
Source: Adv Sci (Weinh). 2025 Nov 19;13(7):e17059. doi: 10.1002/advs.202517059 (PMC12866768; doi:10.1002/advs.202517059)
Supplement: Supplementary file 1 — Supporting Information [file ADVS-13-e17059-s001.docx]

Supporting Information

Bidirectional Photoadaptive Organic Heterojunction Synaptic Transistors for Accurate Image Recognition in Machine Vision Systems

Di Xue, Hongyu Liu, Yingying Zhang, Feng Ding, Jie Lu, Yao Yin, Zi Wang, Jianlong Xu*, Lifeng Chi^*^ and Lizhen Huang^*^

**Figure S1.** AFM height images of diF-TES-ADT films deposited on bare SiO_2_ substrates.

**Figure S2.** AFM height images of template layers deposited on bare SiO_2_ substrates, a) PTCDI-C_13_ films; b) C_32_H_66_ films.


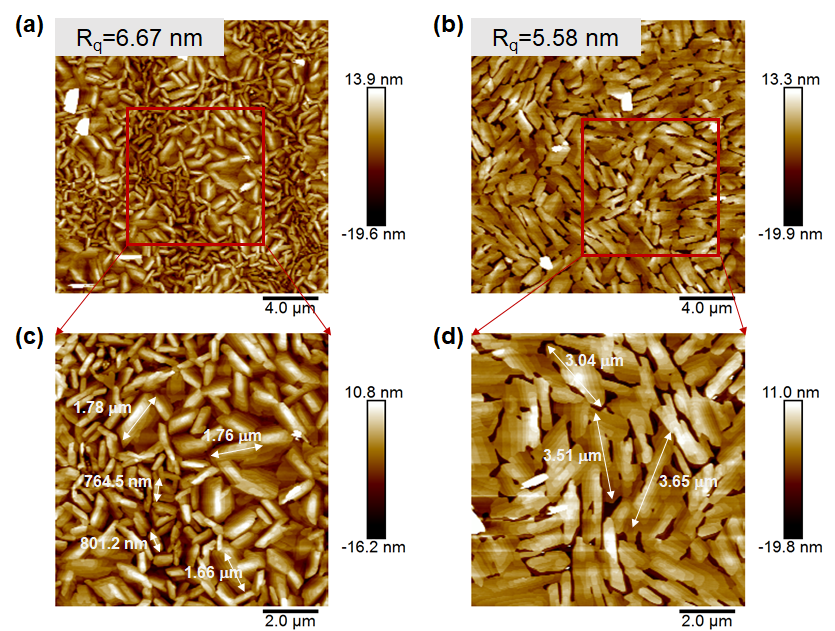


**Figure S3.** AFM height images of diF-TES-ADT-based films, where scanning size are a, b) 20 μm and c, d) 10 μm, relatively, a, c) C_32_H_66_/diF-TES-ADT films, and b, d) PTCDI-C_13_/diF-TES-ADT films.

**Figure S4.** The steady-state photoluminescence (PL) spectra of the diF-TES-ADT-based films, a) excitation wavelength (λ_ex_) = 250 nm; b)λ_ex_ = 365 nm.

**Figure S5.** The typical output characteristics of the diF-TES-ADT-based OPTs, a) diF-TES-ADT OPTs; b) C_32_H_66_/diF-TES-ADT OPTs; c) PTCDI-C_13_/diF-TES-ADT OPTs.

**Figure S6.** Operational stability testing. a, c) C_32_H_66_/diF-TES-ADT OPTs, and b, d) PTCDI-C_13_/diF-TES-ADT OPTs.

**Figure S7.** The comparison of electronical performance diF-TES-ADT OFETs with or without templates, a) *μ*; b) *I*_on_/*I*_off_ ratio; c) *V*_on_. (the error bar depicts the mean ± SD, n = 10)

**Figure S8.** The transfer curves of diF-TES-ADT-based OPTs under series wavelengths (λ = 365 nm, 420 nm, 520 nm, 660 nm, 740 nm) with the fixed illumination intensity (0.35 mW cm^-2^), a) PTCDI-C_13_/diF-TES-ADT OPTs; b) C_32_H_66_/diF-TES-ADT OPTs; c) diF-TES-ADT OPTs.

**Figure S9.** The transfer curves of (a-c) C_32_H_66_/diF-TES-ADT OPTs and (d-f) PTCDI-C_13_/diF-TES-ADT OPTs in dark and under different illumination intensities, a, d) λ = 365 nm; b, e) λ = 420 nm; c, f) λ = 520 nm.

**Figure S10.** a) responsivity (*R*) and b) photosensitivity (*P*) of diF-TES-ADT-based OPTs under different *P*_in_ (λ=365 nm).

**Figure S11.** a-b) Band structure of the PTCDI-C_13_/diF-TES-ADT heterostructure OPTs before and after irradiation when the gate is positive bias, a) in dark; b) under illumination. c) The transfer curves of PTCDI-C_13_/diF-TES-ADT OPTs in dark and under different illumination intensities.

**Figure S12.** a-b) Band structure of the PTCDI-C_13_/diF-TES-ADT heterostructure OPTs before and after irradiation when the gate is negative bias, a) in dark; b) under illumination. c) The transfer curves of PTCDI-C_13_/diF-TES-ADT OPTs in dark and under different illumination intensities.


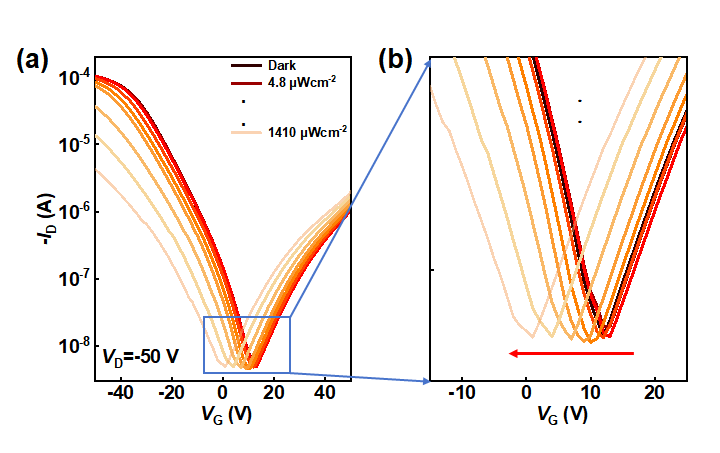


**Figure S13.** a) Transfer curves of PTCDI-C_13_/diF-TES-ADT OPTs in the dark and under different illumination intensities (λ = 365 nm). b) a zoom-in image of the blue frame of Figure 1a.


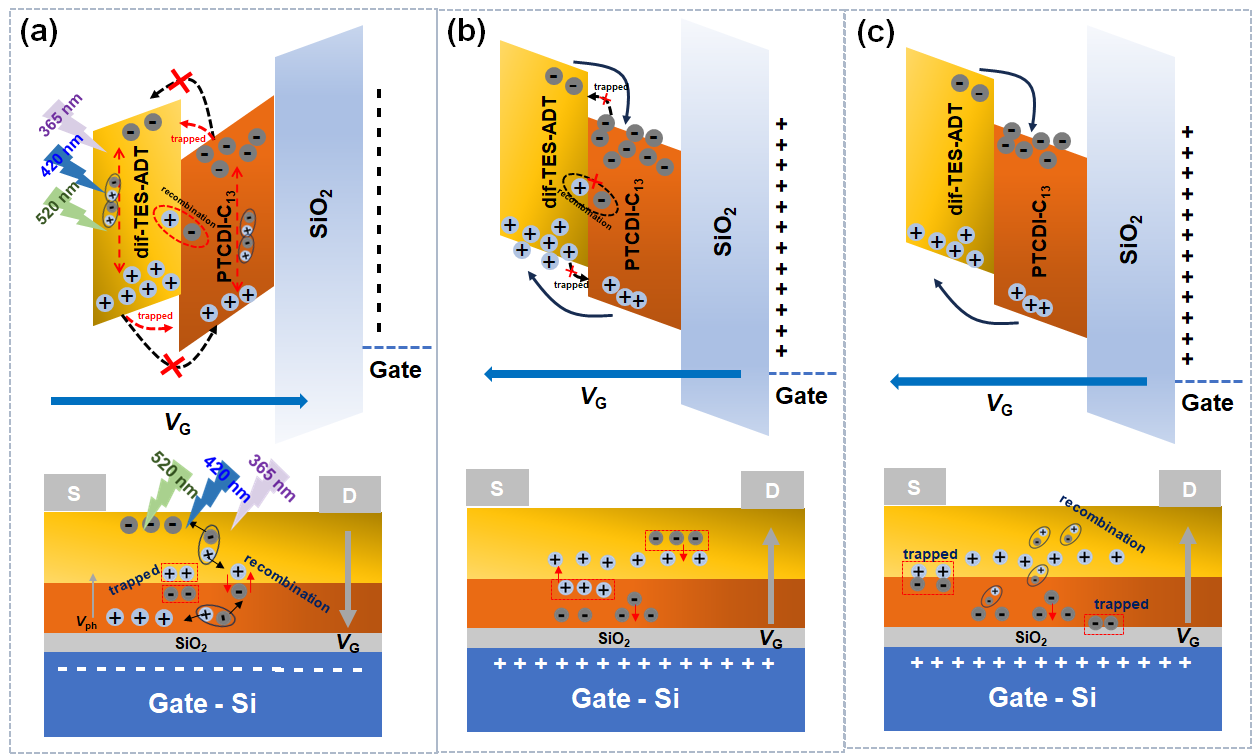


**Figure S14.** Schematic diagrams for the generation, transfer, redistribution process of charge carriers at PTCDI-C_13_/diF-TES-ADT OPTs, a) current decrease upon negative *V*_G_; b) rapid current increase upon positive *V*_G_; c) slow current decay upon positive *V*_G_.

**Figure S15.** Photocurrent curve of diF-TES-ADT-based OPTs when light pulse width (*t*_on_) is 0.3 s, a) C_32_H_66_/diF-TES-ADT OPTs; b) PTCDI-C_13_/diF-TES-ADT OPTs. Here, A commercial UV laser (SIG100) with a wavelength of 355 nm was employed as the incident light source to generate laser pulses with a duration of less than 1 s.

**Figure S16.** The explanation of SW in an artificail synapse. a) EPSC - C_32_H_66_/diF-TES-ADT OPTs; b) IPSC - PTCDI-C_13_/diF-TES-ADT OPTs.

**Figure S17.** Synapse plasticity of the PPC and NPC phototransistors. a, c) The dependence of the transmission from the STP to the LTP on the number of light pulses (λ=365 nm, *P*_in_=700 μW/cm^2^), a) C_32_H_66_/diF-TES-ADT OPTs; b) PTCDI-C_13_/diF-TES-ADT OPTs (*V*_G_= -5 V; *V*_D_= -20 V). b) The changes in SW and characteristic time constants were measured for C_32_H_66_/diF-TES-ADT OPTs by varying the number of light pulses. d) The changes in SW were measured for PTCDI-C_13_/diF-TES-ADT OPTs by varying the number of light pulses.

**Figure S18.** The EPSC and IPSC behavior of diF-TES-ADT-based synapse phototransistors under multiple light pulse cycles (λ=355 nm, *P*_in_=500 μW/cm^2^), a) C_32_H_66_/diF-TES-ADT OPTs; b) PTCDI-C_13_/diF-TES-ADT OPTs (*V*_G_= -5 V; *V*_D_= -20 V). Here, A commercial UV laser (SIG100) with a wavelength of 355 nm was employed as the incident light source to generate laser pulses with a duration of less than 1 s.


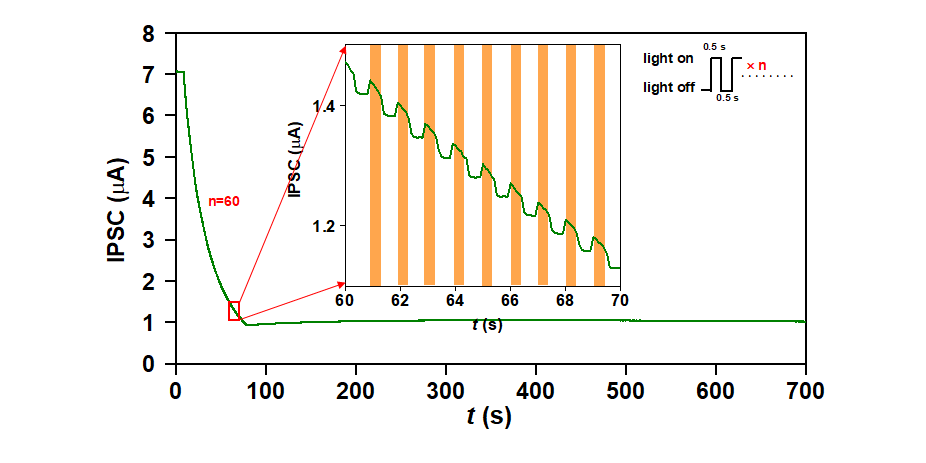


**Figure S19.** The IPSC behavior of diF-TES-ADT-based synapse phototransistors under multiple light pulse cycles (λ=355 nm, *P*_in_=500 μW/cm^2^), where the inset is the zoom-in of red frame.

**Figure S20.** Typical transient photoresponse testing of (a) C_32_H_66_/diF-TES-ADT OPTs and (b) PTCDI-C_13_/diF-TES-ADT OPTs.

**Figure S21.** a, c) Typical transient photoresponse testing of (a) C_32_H_66_/diF-TES-ADT OPTs and (c) PTCDI-C_13_/diF-TES-ADT OPTs under 20 light pulses (λ=365 nm, *P*_in_=700 μW/cm^2^, *V*_G_= -5 V; *V*_D_= -20 V). b, d) the ΔEPSC and ΔIPSC along with the number of light pulses, b) C_32_H_66_/diF-TES-ADT OPTs, d) PTCDI-C_13_/diF-TES-ADT OPTs, a) C_32_H_66_/diF-TES-ADT OPTs; b) PTCDI-C_13_/diF-TES-ADT OPTs.

**Figure S22.** Non-ideal input images treated by traditional image processing algorithms with k values of a) k=0, b) k=0.1 and c) k=0.2.


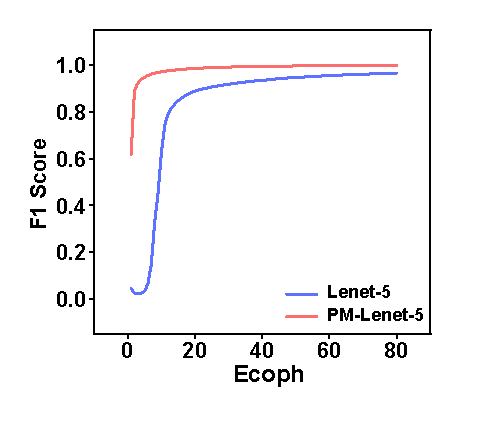


**Figure S23.** The F1-score for the CNN with different architectures.

**Figure S24.** The recognition of MNIST datasets with a) original image, b) without preprocessing, and c) after preprocessed.

**Supplementary Note 1**

These devices are capable of performing adaptive thresholding based on different light intensities, which is particularly useful for preprocessing images with uneven illumination and low contrast. Unlike the conventional devices, we use positive-negative photoconductive devices, which apply both positive enhancement and negative suppression. This enables effective contrast enhancement in bright areas and suppression in dark areas, thereby improving detail in low-contrast regions. The relationship between the adaptive parameter and light power input is written as:

$$f\left( x \right)= a\cdot x^{|m+n|}-b\cdot x^{|m-n|}$$

Where a, b, m and n were the constants extracted from experimental data.

By associating the grayscale value with the corresponding light intensity, the adaptive threshold $T(x,y)$ can be computed from the mean $m(x,y)$ and the standard deviation $s(x,y)$ of the grayscale values within a specified square region:

$$T\left( x,y \right)= m\left( x,y \right)\cdot\left[ 1+k\cdot\left( \frac{s(x,y)}{R}-1 \right) \right]$$

In this equation, *R* represents the dynamic range of the standard deviation for an 8-bit grayscale input image, with *R*=128. The parameter *k* is a correction factor that typically ranges from 0 to 1 in traditional algorithms; however, in this case, *k* is treated as an adaptive parameter dependent on $m(x,y)$, given by:

$$k= 1-f\left( \frac{m(x,y)}{255} \right)$$

To illustrate the effect of the proposed image preprocessing method, the logo image from Soochow University (Figure 5B-A) was utilized. As depicted in Figure 5B, the image, which has uneven lighting and varied contrast, was effectively processed using the positive-negative photoconductive devices, resulting in optimal extraction of features. In contrast, the conventional Sauvola algorithm failed to simultaneously extract both edges and fine details, particularly for low-contrast areas, as shown in Figure S12b,c.

**Supplementary Note 2**

PN‑Lenet‑5 is a device‑informed network in which the bidirectional (positive/negative) photoconductive behaviors of our heterojunction synaptic transistors determine both the preprocessing rule and the dual‑polarity convolutional pathways with an attention gate. We added a detailed flowchart that explicitly ties device behaviors (EPSC/IPSC) to the two convolution branches and to the attention weight that gates them (Figure 8). The diagram shows: (i) photoadaptive preprocessing driven by the device‑extracted parameter k(P_in); (ii) positive and negative convolutional paths mirroring enhancement/suppression; (iii) an attention map A=σ(Conv(X; W_att{K⁺,K⁻})) that prioritizes high‑contrast/illuminated regions; (iv) gated outputs Y⁺_att=A⊙(X∗K⁺), Y⁻_att=(1−A)⊙(X∗K⁻); and (v) combination Y=α·Y⁺_att+(1−α)·Y⁻_att before the standard LeNet‑5 classifier. This figure makes the device→algorithm coupling explicit.

**Figure S25.** The detailed flowchart of machine vision systems combined with PPC and NPC effects.

**Supplementary Note 3**

The positive-negative photoconductive devices process unique enhancement-suppression characteristic, which can be used to design original convolution kernels for feature extraction and iteration.

First, we combine the characteristics of the positive and negative photoconductive devices by defining the convolutional kernels as two sets: and $W^{+}$ and $W^{-}$. As we introduce the attention mechanism, to determine which region is most important, we need to compute the attention weights for each location.

Suppose we have an input feature map ${X\in R}^{C\times H\times W}$, where *C* denotes the number of channels, *H* represents the height, and *W* represents the width. By applying a convolutional layer with a small convolution kernel (often 3×3), we can generate an attention map ${A\in R}^{C\times H\times W}$, where each pixel value indicates the "importance" of the corresponding region.

The formula for generating the attention map is as follows:

$$A=\sigma(Conv(X,{|W}^{+}|+{|W}^{-}|))$$

Where $Conv(X,W)$ represents the convolution operation on the input *X* with a learnable weight matrix formed by $W^{+}$ and $W^{-}$​, and *σ* is the activation function, with common choices including Sigmoid and ReLU. The Sigmoid activation function restricts the attention values to the range [0, 1], representing the weight (importance) of each region.

Once the attention weight map *A* is computed, it can be used to modulate the output of the convolutional kernels. As we have two sets of convolutional kernels, $W^{+}$ for positive photoconductive response and $W^{-}$ for negative photoconductive response, and both kernels have already convolved the input image *X* to produce the outputs $Y^{+}$ and $Y^{-}$, respectively:

$$Y^{+}= W^{+}*X Y^{-}= W^{-}*X$$

Now, using the attention weight map *A*, we can adjust the outputs of these kernels. For example, the positive photoconductive part $Y^{+}$ will be weighted by the attention map *A*, while the negative photoconductive part $Y^{-}$ will be weighted by (1 – *A*), which is the reverse of the attention weight. The weighted outputs are given by:

$$Y_{att}^{+}=A\cdot Y^{+} Y_{att}^{-}=(1-A)\cdot Y^{-}$$

Finally, we combine the weighted outputs of the positive and negative photoconductive convolutional kernels to obtain the final feature map. This can be done through simple addition or weighted summation:

$$Y= {\alpha Y}_{att}^{+}+{(1-\alpha)Y}_{att}^{-}$$

where *α* is a learnable parameter that controls the relative importance of the positive and negative photoconductive convolutional kernels.

**Table S1**

Comparison of recent reported neuromorphic phototransistors

| Materials | λ  (nm) | *R_max_*  (AW^-1^) | *P_max_* | *D***_max_*  (Jones) | Synaptic Function | Neuromorphic Application | Ref. |
| --- | --- | --- | --- | --- | --- | --- | --- |
| CIZS QDs/IGZO | 480-630 | - | - | 3.4×10^15^ | EPSC | Image pre-processing function | ^[1]^ |
| DTT-8/TFT-CN | 365 | 3.8×10^4^ | 5.1×10^2^ | 1.3×10^14^ | EPSC, IPSC, PPF, | Motion target extraction | ^[2]^ |
| β-Ga_2_O_3_ | 250 | 1.01×10^7^ | - | 2.98 × 10^15^ | EPSC | Facial recognition and neuromorphic computing | ^[3]^ |
| Chlorophyll/ PDPP4T | 430 | 2×10^6^ | - | 6×10^15^ | EPSC, PPF, STP-LTP | Simulation of system-level MNIST pattern recognition | ^[4]^ |
| C_10_-DNTT/PCHC | 450 | - | 8.7×10^8^ | 9.42×10^16^ | EPSC, PPF, STP-LTP | Image recognition, high-pass filtering behavior. | ^[5]^ |
| DTT-8 2DMC/CLCN chiral film | 365 | 3.45×10^4^ | 6.96×10^5^ | 3.52×10^15^ | EPSC, PPF, STP-LTP | Information encryption and decryption | ^[6]^ |
| MoS_2_ | 450, 535, 650 | - | - | - | EPSC, PPF, STP-LTP | Color target recognition | ^[7]^ |
| ReS2/CIPS | 405 | -- | - | - | EPSC, PPF, STP-LTP | handwritten digit recognition | ^[8]^ |
| SiC NPs/MoS_2_ | 254 | 1.9×10^4^ | - | 8.4×10^13^ | EPSC, PPF, STP-LTP, LTD | neural  network acceleration | ^[9]^ |
| CsPbBr_3_ QDs/DPPDTT | 450 | 1.92×10^4^ | 3.02×10^7^ | >10^14^ | EPSC, PPF, STP-LTP, LTD | Motion perception | ^[10]^ |
| PEA_2_SnI_4_/PDPP:PC_61_ | Vis-NiR | 1.8×10^4^  (420 nm)  5.7×10^3^  (850 nm) | 4.9×10^5^  (420 nm)  1.9×10^5^  (850 nm) | 1.9×10^16^  (420 nm)  3.3×10^14^  (850 nm) | EPSC, PPF, STP-LTP | Visual adaptive behavior | ^[11]^ |
| C_32_H_66_/diF-TES-ADT | 365-520 | 1.40×10^4^  (365 nm) | 5.73×10^6^  (365 nm) | 1.74×10^16^  (365 nm) | EPSC, IPSC, PPF, PPD, STP-LTP | Human visual photoadaptation functionality mimic, low-contrast image recognition | This work |
| PTCDI-C_13_/diF-TES-ADT | 365-520 | 721.15 (NPC)  (365 nm) | 0.98 (NPC)  (365 nm) | 3.7×10^12^ (NPC)  (365 nm) |  |  |  |

[1] T. Chen, S. Zhan, B. Li, B. Hou, H. Zhou, A Low‐Toxic Colloidal Quantum Dots Sensitized IGZO Phototransistor Array for Neuromorphic Vision Sensors. *Adv. Opt. Mater.* **2024**, *12*, 2302451.

[2] X. Zhu, C. Gao, Y. Ren, X. Zhang, E. Li, C. Wang, F. Yang, J. Wu, W. Hu, H. Chen, High-Contrast Bidirectional Optoelectronic Synapses based on 2D Molecular Crystal Heterojunctions for Motion Detection. *Adv. Mater.* **2023**, *35*, e2301468.

[3] X.-X. Li, G. Zeng, Y.-C. Li, Q.-J. Yu, M.-Y. Liu, L.-Y. Zhu, W. Liu, Y.-G. Yang, D. W. Zhang, H.-L. Lu, Highly Sensitive and Stable β-Ga2O3 DUV Phototransistor with Local Back-gate Structure and its Neuromorphic Application. *Nano Research* **2022**, *15*, 9359.

[4] B. Yang, Y. Lu, D. Jiang, Z. Li, Y. Zeng, S. Zhang, Y. Ye, Z. Liu, Q. Ou, Y. Wang, S. Dai, Y. Yi, J. Huang, Bioinspired Multifunctional Organic Transistors Based on Natural Chlorophyll/Organic Semiconductors. *Adv. Mater.* **2020**, *32*, e2001227.

[5] M. Ding, T. Jiang, B. Wang, Y. Li, J. Zhang, J. Huang, D. Ji, W. Hu, Environmentally friendly and degradable organic neuromorphic vision sensors. *Matter* **2024**, *7*, 1736.

[6] Y. Zhang, M. Dong, Y. Du, S. Yang, Y. Ren, Y. Guo, D. Gao, X. Lin, D. Yuan, G. Zhou, Y. Yan, L. Sun, R. Li, F. Yang, W. Hu, Chiroptical organic heterojunction synaptic phototransistor exhibiting near-theoretical limit asymmetry factor for neuromorphic cryptography. *Matter* **2025**, *8*, 101945.

[7] B. Wei, Y. Chen, X. Han, Y. Kang, B. Liang, C. Li, X. Yang, L. Fang, Y. Peng, Ultra-low Power MoS2 Optoelectronic Synapse with Wavelength Sensitivity for Color Target Recognition. *Science China Information Sciences* **2025**, *68*, 140406.

[8] K. Zhang, Z. Huo, Y. Wang, H. Yan, J. Yu, L. Cheng, Y. Fu, C. Liu, Z. Li, Z. Liu, Z. L. Wang, Q. Sun, X. Lin, Neuromorphic Tactile-visual Perception based on 2D ReS₂/CIPS Heterojunction Artificial Synapse. *Nano Energy* **2025**, *144*, 111399.

[9] Z. Lian, J. Wei, Y. Liu, Z. Liu, Y. Liu, M. Xie, Y. Dan, C. C. Tu, R. Yang, Highly Responsive Dual-Function Deep-Ultraviolet Neuromorphic Phototransistors Based on Silicon Carbide Nanoparticle/2D MoS(2) Heterostructures. *ACS Nano* **2025**, *19*, 26041.

[10] Y. Wu, S. Dai, X. Liu, P. Guo, J. Zhang, T. Sun, Z. Guo, Y. Xu, H. Liang, L. Xiong, H. Hu, J. Huang, Optical Microlithography of Perovskite Quantum Dots/Organic Semiconductor Heterojunctions for Neuromorphic Photosensors. *Adv. Funct. Mater.* **2024**, *34*, 2315175.

[11] X. Huang, M. Wang, W. Wen, S. Wei, K. Zhang, Y. Guo, Y. Liu, Constructing Perovskite Organic Phototransistors Using a Triple Strategy to Achieve Visible and NIR Visual Synapses and Adaptive Functions. *Small* **2025**, *21*, e2412025.
